# Decisions to use complementary and alternative medicine (CAM) by male cancer patients: information-seeking roles and types of evidence used

**DOI:** 10.1186/1472-6882-7-25

**Published:** 2007-08-04

**Authors:** Maggie Evans, Alison Shaw, Elizabeth A Thompson, Stephen Falk, Pat Turton, Trevor Thompson, Deborah Sharp

**Affiliations:** 1 Academic Unit of Primary Care, Department of Community-based Medicine, University of Bristol, 25-27 Belgrave Road, Bristol BS8 2AA, UK; 2 Bristol Homeopathic Hospital, Cotham Hill, Bristol BS6, UK; 3 Bristol Haematology and Oncology Centre, Horfield Road, Bristol, BS2 8ED, UK; 4 University of the West of England, Coldharbour Lane, Bristol BS16 1QY, UK

## Abstract

**Background:**

Complementary and Alternative Medicine (CAM) is increasingly popular with cancer patients and yet information provision or discussion about CAM by health professionals remains low. Previous research suggests that patients may fear clinicians' 'disapproval' if they raise the subject of CAM, and turn to other sources to acquire information about CAM. However, little empirical research has been conducted into how cancer patients acquire, and, more importantly evaluate CAM information before deciding which CAM therapies to try.

**Methods:**

Qualitative study, comprising semi-structured interviews with 43 male cancer patients of varying ages, cancer type and stage of illness, 34 of whom had used CAM. They were recruited from a range of NHS and non-NHS settings in Bristol, England.

**Results:**

As a result of the lack of CAM information from health professionals, men in this study became either 'pro-active seekers' or 'passive recipients' of such information. Their main information resource was the 'lay referral' network of family, friends and acquaintances, especially females. 'Traditional' information sources, including books, magazines, leaflets and the media were popular, more so in fact than the internet. Views on the internet ranged from enthusiasm or healthy scepticism through to caution or disinterest. CAM information was generally regarded as 'empowering' as it broadened treatment and self-care options. A minority of participants were information averse fearing additional choices that might disrupt their fragile ability to cope. There was general consensus that CAM information should be available via the NHS, to give it a 'stamp of approval', which combined with guidance from informed health professionals, could help patients to make 'guided' choices. However, a small minority of these men valued the independence of CAM from the NHS and deliberately sought 'alternative' information sources and treatment options.

Men were selective in identifying particular therapies to use and sceptical about others, basing their choices on forms of 'evidence' that were personally meaningful: personal stories of individuals who had been helped by CAM; the long history and enduring popularity of some therapies; the plausibility of the mechanism of action; a belief or trust in individual therapies or their providers; scientific evidence. Scientific evidence ranked low in the men's personal decision-making about CAM, while it was recognised as important for NHS support for CAM.

**Conclusion:**

These male cancer patients valued the support and guidance of 'trusted individuals' in making choices about CAM. Trusted health professionals could also play a significant role in helping patients to make informed choices. Any such dialogue must, however, acknowledge the different standards of evidence used by patients and clinicians to evaluate the benefits or otherwise of CAM therapies. Such open communication could help to foster an environment of mutual trust where patients are encouraged to discuss their interest in CAM, rather than perpetuate covert, undisclosed use of CAM with its attendant potential hazards.

## Background

The increasing use of complementary and alternative medicine (CAM) by cancer patients has been well documented [1,2] and yet the reported level of disclosure to medical practitioners (33% – 60%) remains of concern, in view of the risk of potentially harmful pharmacological interactions [3]. A culture of 'pervasive silence' and 'professional disinterest' has been described in relation to CAM, so patients may be reluctant to raise the subject in case health professionals 'disapprove' [4]. Clinicians and nurses are rarely cited as a source of information about CAM [2], leaving the field wide open for patients to explore other, potentially less reliable, avenues. For example, much has been written about the increasing popularity of the internet as a source of health information [5], allowing unregulated access to 'unproven' and potentially harmful treatments [6]. Little empirical research has been conducted, however, on what sources of CAM information cancer patients actually use. Some studies have suggested that, despite the advent of the internet, 'traditional sources' of information are still important to cancer patients seeking disease-related information [7] and that preferences for different forms of information may be related to ethnicity and educational level [8]. With regards to internet use, one author found that patients displayed 'considerable caution and competence' in handling information about their condition obtained in this way [9]. We do not know whether the same applies to patients' handling of information about CAM therapies.

There is also little understanding of how CAM information is used and evaluated by patients in their decisions whether to try CAM and which therapies to use. Patients may be using evidence based on very different notions of 'therapeutic efficacy' compared to clinicians [10]. Research suggests that patients tend to use 'anecdotal' rather than 'scientific' evidence and this may impact on the ability of professionals and patients to 'develop a dialogue about CAM decision making' [11].

This study focuses on use and evaluation of CAM related information by male cancer patients. It draws on qualitative data from a larger study of male cancer patients' attitudes towards and use of CAM. Previous studies have shown that male cancer patients tend to request support and information about their condition less than women, both within consultations and from other sources [12,13]. Thus it is important to investigate what sources of information are preferred by men, and whether, in fact, the reported popularity of the internet has impacted on their knowledge about CAM. This paper focuses particularly on how male cancer patients obtain, evaluate and use CAM information, drawing out some implications for information provision and patient-professional communication.

## Methods

A qualitative research design was used in order to explore the processes involved in gathering and evaluating information about CAM from the perspective of men with cancer. Ethical approval was gained from the local NHS Research Ethics Committee.

### Participants

Purposeful sampling [14] was used with a recruitment strategy aimed at maximum variation with regards to age, cancer type, stage of illness and potential attitudes towards CAM. In order to identify men who potentially held a range of views about CAM, they were recruited from three contrasting settings: an NHS oncology centre (where CAM users were identified in outpatient clinics using a short screening questionnaire), the outpatients clinics at a NHS homeopathic hospital and a cancer charity providing CAM therapies paid for privately. A small sub-sample of patients who had never used CAM was also recruited.

### Data collection

Semi-structured interviews were carried out with participants in their own homes, covering a range of topics relating to their use of CAM. The topics reported here include: how information on CAM was obtained; which sources of information were most useful; how this information was used in decisions about using CAM. Interviews were recorded and fully transcribed and the data were analysed thematically, drawing on the principles of constant comparison [15].

### Data analysis

ME took the lead in coding and analysing the data, aided by the qualitative analysis software Atlas.ti. AS read and coded a sub-set of the interview transcripts from each recruitment setting, and met with ME to discuss the developing coding framework and agree on broad themes. Members of the steering group also read sections of some transcripts and commented on the coding framework.

## Results

### Participants

The study comprised thirty-four male cancer patients who were using CAM, evenly distributed across the treatment sites: eleven from the oncology unit, twelve from the homeopathic hospital and eleven from the cancer charity. A broad spectrum was achieved across the sampling criteria. The age of participants ranged from 31 to 83 years, with a mean of 57 years. The spread across cancer type and stage of illness is shown in Tables 1 and 2. Participants were white and came from a range of manual, non-manual and professional occupational backgrounds. Over half were professional. Twenty-two participants had used CAM before their cancer diagnosis, for other health problems or for health maintenance, while for twelve participants, cancer had been the trigger to CAM use. Seven 'non-users of CAM' were also interviewed and they cited lack of knowledge and information about CAM as major reasons for non-use. Some non-users were sceptical about CAM but felt that CAM information and advice should be more readily available through the NHS.

**Table 1:** Cancer type

**Type of cancer**	**No of participants**
Colo-rectal	10
Prostate	10
Lung	3
Other (thymic, tonsillar, pancreatic, bone, lymphoma, bladder, renal, oesophageal, leukaemia)	11

**Table 2:** Stage of disease

**Stage of disease**	**No of participants**
Localised	10
Remission	10
Metastatic	8
Palliative care	6

This paper will focus on those men who were using CAM. The types of CAM used by the men are shown in Appendix 1. All participants used CAM alongside their conventional treatment, except for one man who had refused orthodox treatment in favour of CAM therapies. The ways in which CAM was used and the perceived benefits are reported elsewhere [16]. The focus of this paper is how the men sought, evaluated and used CAM information. Data illustrating each theme are mostly provided in tables 4 to 9, with some quotes illustrating key points interspersed within the text.

### Information resources used

Men talked about the lack of CAM information available from the NHS and the difficulty in engaging in discussion about CAM with professionals they encountered during their conventional care (see Appendix 2). One man, who went on to access support from the CAM charity, described his frustration and sadness. 'I asked my oncology doctor are there any other avenues and the response was well there are one or two things but nothing has been proven and there's nothing very definite ... and I suppose that's what's I suppose is very sad ... I chose not to probe ... you can detect there is no inclination, no desire, no WANT to debate it or discuss it ... you get this very clear message' *[age 64, oesophageal cancer in palliative care]*. Another participant talked about the possibility of empowering patients by making CAM information accessible. 'I just think that information should be out there for people to make decisions and to let them know that it's there because even just on a pain level, because there's there are therapies out there which can help people with their pain management and if you can manage your pain that helps your process so much' *[age 32, recurrent bone cancer]*.

The main resource for these men was the 'lay referral network' [17]: personal stories and recommendations passed on by family, friends, work colleagues and other acquaintances, often provoked by a cancer diagnosis. Some responded to advertisements in local shops. Female family members played a significant role in steering men towards CAM, especially wives and daughters who had knowledge or personal experience of CAM (see Appendix 3). For example, this man reflected on the important role his daughter had played. 'I was lucky with my daughter .... How I would have done without her .... because she's been a nutritionalist for some time .... she's always been a health enthusiast but she's a realist, she believes in really a healthy diet and she doesn't mean filling you up with vitamins' *[age 74, colo-rectal cancer in remission]*.

The internet was rarely the first port-of-call for CAM information (see Appendix 4); in fact it was used more often to research conventional rather than CAM treatments. With regards to CAM, the internet provided the means to investigate a CAM therapy mentioned by an acquaintance, to order books or nutritional supplements or to find a CAM therapist via a professional organisation. Those who used the internet described how they were selective in their choice of web-sites, rather than pursuing general searches, as illustrated by this man, who stopped searching 'once I found the charity web-site ...which was very good because a lot of the people who were there have had the disease and a lot of the information is from people who've been through the same thing' *[age 54, prostate cancer in remission]*.

A surprising proportion of the men lacked either the experience or the inclination to engage in internet searching and this was true across all ages. 'No I have not [used the internet] ... I have looked I mean there are dangers there I mean quite apart from all the trash that comes in trying to sell us Viagra and all sorts of things .... So no no I have not, I am very happy to take the advice and treatment offered by [holistic doctor] for example and the acupuncture as well!' *[age 73, prostate cancer in remission]*

Some of the older participants described how searches had been performed on their behalf by family members, such as this man: 'The internet, my wife's a bit of a dab hand on the internet and that, she's got an awful lot of information ... there's so much of it on there you know it sort of gives you some sort of hope when everything seems lost....

Q: Have you actually ordered anything over the internet?

No ... oh well the bush telegraph I mean is better than the internet really' *[age 60, locally advanced lung cancer]*.

'Traditional' sources of information such as books, leaflets and the media were generally more popular than the internet (see Appendix 5). For example men responded to written information in the form of booklets provided by charities, Yellow Pages and mailshots. 'How I chose the ones [types of CAM] that I'm pursuing I went by this little book particularly to start with, having read the cancer BACUP book which listed them and a paragraph or two about each one .. and this book, the 'Which Guide to Complementary Therapies', I think I bought it from Waterstone's or somewhere like that ... and 'The Web that has no weaver' it's an attempt in some ways to reconcile Chinese and Western medicine' *[age 65, metastatic colo-rectal cancer]*.

Stories about CAM and its proponents were frequently found on television and radio or read in newspapers and magazines. Books recounting stories of survival from cancer, or advocating anti-cancer diets and CAM therapies were passed around the 'lay network'. 'The friend from Australia sent me some tapes, meditation, visualisation tapes and a book by I.G. who's beat cancer in his own way, and I'm just interested to read his story, so I started off listening to these tapes and realising there's something in this, so I thought I need to go a little bit more in that direction so I went to the [private cancer charity]' *[age 49, metastatic colo-rectal cancer]*.

Two key themes regarding information use emerged in the analysis. Data such as these, that are based on personal experiences, serve as an illustration of how a group of patients navigated their way through the CAM information available, some of which has been referred to as 'misleading' and potentially 'harmful' [6]. The two main themes are as follows: First we will consider the role patients take in information seeking – whether they are pro-active seekers or passive recipients of CAM information. Second we will consider the types of evidence and criteria that patients use to evaluate CAM. The themes presented are supported by data reflecting the full range of expressed views (see Appendices 6 and 7).

### Theme 1: Patients as pro-active seekers or passive recipients of CAM information

A willingness on the part of these men to be informed about CAM was linked to their desire to know as much as possible about their illness generally, including their conventional treatment options. Participants could be broadly categorised into those who actively sought out information about CAM (pro-active seekers) and those who took a more passive stance and simply picked up information that came their way (passive recipients) (see Appendix 6). Which role they took depended partly on their cancer type and stage of illness. Pro-active information seekers were often men with more rare cancers, fast-growing tumours or advanced disease. For these men, information about CAM included seeking out 'alternative' and potentially 'curative' treatments, especially if conventional treatments could no longer help them. Some men who were at earlier stages of illness or who had more common types of cancer, also took a pro-active approach but they more commonly searched for information about supportive or complementary treatments to be used alongside their conventional treatment.

Men's role in information gathering also related to the stage at which they had started using CAM. The men in this study fell into two broad groups: those who had used CAM prior to their cancer (the majority) and those for whom a cancer diagnosis was the trigger to CAM use. Those characterised as pro-active seekers of CAM were more likely to have already used CAM before their cancer diagnosis than to be 'new' users. 'Pro-active seekers' tended to embrace a wider range of therapies than those who were 'passive recipients' of information.

Those who could be characterised as 'passive recipients' of CAM information (just over one third of the participants) were equally divided between those who had used CAM for previous health problems and those who were 'new' users following their cancer diagnosis. CAM information was generally welcomed and acted upon, even if it was treated initially with scepticism. Passive recipients were (perhaps unsurprisingly) more likely to be drawn from the NHS recruitment sites rather than the private setting, and a few had in fact first heard about CAM from an NHS health professional, albeit in a very general way. They recounted how CAM therapies were sometimes suggested by a doctor or nurse in primary care, both prior to and since the cancer diagnosis: for example, a recommendation to try chiropractic for a back problem or homeopathy for cancer-related symptoms. Within oncology services, however, CAM was mentioned only occasionally in a non-specific way as a possible support for end-stage patients, but with no specific recommendations. As a result of these suggestions, in combination with encouragement by family members who had some experience or knowledge of CAM, some men were triggered into CAM use. However, in contrast to the pro-active information seekers who embraced a wide range of therapies, those with a more passive role in acquiring information tended to use only the therapy that they first encountered, for example taking nutritional supplements or attending the homeopathic hospital.

#### Satisfaction with information-seeking role

Participants reflected on their role in information gathering, some feeling that they had taken on either a pro-active or a passive role by choice, but others noting how they took up their position rather by default, because they felt it was their only option. For example, some described how the passive role sometimes arose as a result of the lack of open discussion about CAM within the NHS and the difficulty they experienced in raising the subject. Some speculated as to whether oncologists' reluctance to engage with the topic was driven by individual disinterest or scepticism, or by hospital policy.

Others, however, responded to the lack of CAM information available from NHS health professionals by taking on a pro-active information-seeking role. 'He [oncologist] was a bit offhand, wasn't really ready to give me much information, I had to sort of, you know, pump him shall we say .... I got the information out of him but it was a question of sort of pulling teeth if you like, if you didn't ask the question you didn't get told was the impression I got' *[age 52, localised prostate cancer]*. Some willingly took on this role, whereas others found it difficult on several counts. First: they found it difficult to formulate questions about CAM within consultations, not knowing what or who to ask and what clinicians' reactions would be. Second: they were reluctant to go outside of conventional medicine to seek information and support regarding CAM, having the hope and expectation that CAM information would be more easily available within the NHS and expressing disappointment when it was not. Third: they wanted a stamp of approval from NHS professionals regarding the CAM therapies they chose to use, preferring those that would be seen as medically 'legitimate'.

As a minimum, many of the men felt that NHS professionals should adopt a 'sign-posting' role, even if they did not provide detailed information on CAM, for example by providing a list of CAM information resources and practitioners. Amongst the pro-active information seekers there was also a minority who preferred to look outside the NHS for CAM information and were seeking 'alternative' approaches to their illness and its treatment.

#### Information as empowering or anxiety provoking

Whether men took a 'pro-active' or 'passive' approach to gathering CAM information may partly be explained by their underlying general attitude towards information about their condition. 'Pro-active' seekers generally regarded information as a tool for empowerment, whereas the more 'passive' approach was typical of men who perceived information as potentially unsettling or anxiety provoking. 'Empowerment' was experienced as getting a better understanding of the illness and its treatment that enabled participants to make informed contributions to treatment decisions or to engage in self-care. 'I said thank you very much that's great and I've been really wanting this information, I'm not ready for an operation yet, I feel good, I'm not ready for one, I'm thinking of moving and he was a bit put out at that, this person, given all this information, and he doesn't want, he said well OK he said I really advise that we should operate as soon as possible before it gets too big' *[age 43, recurrent bone cancer]*.

Some wanted very detailed information such as survival statistics, personal prognosis, tumour grading, details of surgical procedures or wished to view their scans. They also sought information about psycho-social support, to help them deal with either living or dying with their disease. CAM information in particular was perceived as 'empowering' as it led to opportunities for self-care and gave feelings of control and hope in a potentially fearful and life-threatening situation. Such information broadened their treatment and supportive care options in ways that they weren't routinely getting in their NHS care. For many, finding out about CAM was a strategy to fill perceived gaps in their conventional care [16].

Amongst the 'passive recipients' of information were some who were wary of finding out too much about their condition for fear that the information would bring with it added anxiety, responsibility and stress, with further decisions to be made. They had clear boundaries around what they wanted to know and reported feeling overwhelmed if confronted with 'too much' information. They viewed information as 'unsettling' with the potential to disrupt their fragile ability to cope and they preferred to focus on what lay immediately ahead of them. Information brought to them by well-wishers could be seen as an unwelcome intrusion. 'The worst for me was people bringing me sheets of paper [from the internet] at work and you read them and think I'm not going to go through that and there was so much ... trouble is you read 'Cancer' and all you read about is people dying, the local paper are doing a series about it, one in four people getting over it, four out of ten are going to die and that's what the internet is it's instantly that you go in and you think you're better off getting treated in Australia or there's America, and its pages long' *[age 54, prostate cancer in remission]*.

#### Changes in information-seeking role

The boundaries between the categories of 'passive recipient' and 'pro-active seeker' were inevitably fluid and there was evidence of change over time as men's interest in CAM grew and their approach to acquiring information evolved over time. Some of those who were initially 'passive recipients' of CAM information shifted roles to become 'pro-active seekers' as they acquired information about CAM and developed an interest in finding out more. Some men who were initially sceptical about CAM changed their views and adopted CAM as part of their overall treatment and support strategy. They described a growing awareness of stories and issues relevant to cancer and its treatment (both conventional and CAM) as they lived with their condition. Others found their interest in CAM initially stimulated by the chance reading of a magazine article or an inspirational book lent by a friend which then triggered them into a more active role in finding out about CAM.

Once men started actively seeking CAM information, a process of 'snowballing' took over as networks of resources were uncovered. 'A lot of books, research on the internet, all kinds of articles, as soon as you've got cancer you come across other people who know about other people and relatives and the like who've been through it all and they say oh have you seen this book, seen this article, look at that website, you end up with a mountain of information' *[age 51, tonsillar cancer palliative care]*.

### Theme 2: Types of evidence: patients' criteria for evaluating CAM information and therapies

Patients used a variety of forms of 'evidence' to make judgements about CAM and whether they would use a particular therapy. Rather than a wholesale acceptance of all types of CAM, the men in this study described how they attempted to be discerning in evaluating the information available, in order to choose the type of CAM that might suit them. They were usually very selective in identifying a particular CAM for a particular problem and were often sceptical about other therapies. For example many men were wary about any form of therapy that involved participation in a group, one man expressing this as distaste for 'hand-holding and all that sort of thing'. Within the array of information available to them, patients tended to use forms of 'evidence' that made sense to them and were personally meaningful: often quite different from the types of evidence traditionally valued by clinicians (see Appendix 7). The types of criteria and evidence that patients drew upon to evaluate CAM included the following:

#### Personal stories

Participants were particularly impressed by the evidence of personal stories about individuals, including family members, who had been helped by CAM, often for complaints other than cancer. Biographies of cancer survivors who had used CAM were regarded as inspirational, mainly as a model for personal self-help rather than giving patients belief in a 'cure'. 'She was diagnosed with breast cancer and ... decided to do something about it and therefore looked at the total bit not only her mental attitude but her diet her exercise, did everything, meditated, got in touch with her spirituality did absolutely everything that she could do to make herself better to rebuild her immune system and to fight the disease and she was successful doing that for twenty years' *[age 43, lung cancer in remission]*. Such stories were sourced in books, the internet, leaflets, newspapers and magazines. Some regarded their own story of survival to date as 'living proof' of the effectiveness of CAM in combination with conventional treatment. Other pointed to specific benefits they were experiencing themselves as sufficient evidence of the effectiveness of their chosen CAM therapy.

#### History

A common criterion for judging the quality and likely effectiveness of a CAM therapy was its history. The long history and enduring popularity of many therapies, for example herbal remedies and acupuncture, were often perceived as indicators that they benefited patients. Participants were also more inclined to view CAM therapies positively where there had been a history of using herbal or other 'natural' products over many generations within their own family. 'I used to have cod liver oil and malt as a kid I remember my mother ...but that [supplement] does help your joints definitely ... there was no proof I don't think in those days, you just you did that you know, if you sting yourself, rub a dock leaf on it ...one shouldn't say a treatment is no good because it hasn't been scientifically proved' *[age 83, locally advanced prostate cancer]*.

#### Plausibility

Participants gave weight to the 'plausibility' of a CAM therapy as discerned from the information they had gathered and they would only try a therapy that seemed plausible in its mechanism of action. This was very much an individual judgement and depended on their conceptualisation of the particular therapy. For example some had no belief in the proposed mechanisms of action for acupuncture, while others believed it might work via the nervous system and others believed it worked via the flow of energy. Where people had a belief in the power of the mind to effect physiological change, they might be more inclined to accept therapies that had a 'mind-body' dimension, for example visualisation, meditation or hypno-therapy. 'Relaxation, hypnotherapy side of things which again seemed to be suggesting I mean they're all different names for the same thing meditation, relaxation, hypnosis, that the sub-conscious can in fact have quite profound effects on physiological processes' *[age 65, metastatic colo-rectal cancer]*.

Men with a scientific background found it easier to accept the use of herbal remedies or nutritional supplements, likening them to allopathic drugs synthesised from natural products. One scientist was convinced that homeopathy and acupuncture were plausible, based on evidence he had read regarding their effectiveness in animals, thus implying that the effects were more than placebo.

#### Belief and trust in therapies and their providers

While the majority of participants were using CAM outside the NHS, they often expressed a preference for CAM to be provided via the NHS since this is a trusted source of care and brought with it a 'stamp of approval'. Some requested referral to the NHS homeopathic hospital, which was made easier where they had a prior relationship with a GP with an interest or training in CAM. Such GPs became a resource for CAM information and referral. In the absence of NHS endorsement, participants placed their trust in CAM therapists who had recognised qualifications and professional registration, or who worked at well-known centres. 'If they did advertise or there was leaflets in hospitals then at least they could have a maybe a list of people what have been checked out or that they know they've done the qualifications and the certificates are alright and not just someone, you see it so often on the telly about these fake doctors and everything that just print their own certificates' *[age 43, locally advanced colo-rectal cancer]*.

Enduring engagement with a CAM provider, however, depended more on the personal qualities and experience of the provider and the therapeutic relationship than on their formal qualifications.

The same desire for seeking 'approved therapies' was evident in the way participants sought out information from the internet. The majority approached the internet with caution, choosing only to visit medical, professional or charity websites and steering clear of more commercial sites. 'I think one of the things you've got to realise is that whoever's web page you're on is probably promoting their product ... you can sort of dodge around and look at various sites, cross-reference them so, yeh so it is useful' *[age 61, prostate cancer in remission]*.

However, professional authentication was not the only 'quality label' that impressed would-be CAM users. Therapies were seen as legitimate or credible if recommended by trusted individuals, be they friends, family members or CAM therapists. Some preferred to trust their own inner judgement and relied less on external markers of credibility, describing for example how they acted 'in faith' or relied on 'gut instinct'. A minority actually valued the independence of private CAM provision from the NHS, expressing preference for the 'alternative' philosophical approaches that under-pin some therapies. Despite this orientation towards more alternative approaches, these men too talked about using the internet in a discerning way and were critical of websites giving treatments that did not seem credible to them.

#### Scientific evidence

A common belief among the men studied was that lack of scientific evidence did not equate with lack of effectiveness. On this basis, they were prepared to give CAM therapies the benefit of the doubt. Some men, notably those from a scientific background, reflected on methods used to study CAM and conventional treatments, identifying what they saw as the limitations of conventional drug trial evidence. Their concerns were that such trials report average rather than individual effects and can under-estimate long-term safety considerations. Several expressed the belief that scientific methods were not always appropriate to evaluate CAM since some therapies pre-dated the advent of the scientific approach and are based on radically different philosophies and mechanisms of action. Whilst recognising that scientific evidence of effectiveness would be beneficial to attract funding or support for CAM within NHS, the lack of such evidence did not stop them personally using CAM. 'I don't think I'm bothered by the scientific proof about them but I would be willing to try them, it's maybe the person who's dispensing them I need to know that they're ... I would prefer to do that than just try things willy nilly go round trying things, I need to try things for a reason' *[age 56, non-Hodgkin's lymphoma in remission]*. The benefits of some therapies, such as diet and relaxation, were seen to be common sense, thereby excluding the need for 'scientific proof'. Scepticism about the influence of the pharmaceutical companies in promoting certain drugs and marginalising 'alternative' treatments was common.

## Discussion

The men in the study said they would welcome more open discussion and advice about CAM from health professionals. In contrast, their experience reveals that little CAM information was available to them via the NHS and they found it difficult to broach the subject of CAM with health professionals. Men felt that the onus was on them, the patients, to inform themselves and make their own decisions about whether or not to use CAM and some felt uncomfortable about taking on such a pro-active role. This situation may not, however, be unique to CAM. Our data support earlier findings that doctors tend to give information about illness and treatment only to patients who actively request and seek it [18] thus reinforcing the pro-active role required of patients.

Against this background of a perceived reluctance on the part of doctors to give out additional information willingly, patients are likely to have a particularly difficult time finding out about CAM, a subject about which doctors themselves may have limited knowledge or interest or about which they may hold views which are at variance with those of their patients. As reported elsewhere, this may impact negatively on patients' ability to cope with their cancer, since those men who did successfully access CAM regarded it as a crucial component of their supportive care [16].

Many participants did feel comfortable with a pro-active role as a CAM information seeker and indeed, previous studies show how CAM users frequently benefit from the sense of personal 'empowerment' that accessing CAM can provide, with its opportunities for consumer choice and its emphasis on self-care [19,20]. Our study suggests, however, that there are also a group of patients who prefer a more 'passive' role and would prefer their clinician to provide advice or make decisions for them. Amongst the participants characterised as more 'passive', many were in fact open to and welcomed the provision of information and advice about CAM from trusted individuals. Such individuals at present tend to consist of partners, family and friends, who have been shown to play a significant part in influencing decisions about CAM use in this and other populations of cancer patients [21]. Most participants in the present study clearly saw health professionals as trusted individuals and so, equipped with appropriate information, they too could play a significant role in helping patients make informed choices about CAM use. The growing number of specialist nurses in oncology comprises a professional group that might feasibly take on this role. Another option might be for such a role to be taken on within primary care, either by doctors or nurses, especially since the registration and follow up of cancer patients are targets for GPs in the new contract [22,23].

Patient choice, participation in decision-making and the 'expert patient' [24] are all concepts endorsed by current NHS policy, yet studies such as this indicate that the translation of concepts of choice from policy into practice is far from complete. One cause of this may be a potential tension between the evidence-based medicine and patient choice agendas that both underpin NHS policy, as has been recently highlighted in a study of asthma patients use of complementary therapies [25]. The present findings also contribute to the broader debate about the changing nature of the doctor/patient relationship from the traditional 'paternalistic' model to the alternative models of 'shared decision-making' or the 'consumerist' approach where patients take greater responsibility for informing themselves and being involved in treatment decisions. It may be that the balance has gone too far towards placing the onus on patients to be informed – a finding that concords with studies in other areas of patient care [26]. Despite a recent policy emphasis on encouraging patient choice [27], too much choice can be confusing for some patients. With regards to CAM, patients may need guidance and help in making decisions and NHS professionals could develop a stronger role in helping patients to make appropriate choices about CAM use. This is graphically illustrated in this quote: 'Imagine being in a fast flowing river and the guy on the bank has got half a dozen different aids to help you and he's shouting to you which one do you want? You know, I don't care which one it is as long as it, you know don't you know which one to throw *'[age 61, prostate cancer in remission]*.

A recent study of health information seeking (not specifically about CAM) showed that women with breast cancer adopt similar strategies to the men in this study in order to inform themselves about their illness and its treatment [7]. They, too, fell into distinct groups, some taking an active approach to information gathering and others preferring a more passive approach. The women, like the men in the present study, also stated a preference for traditional print media rather than electronic media as a form of communication. In view of concerns about the lack of regulation of e-information [28,29], it is important to note that, in both studies, information picked up from the popular press or from books written by cancer survivors, was used in CAM decision-making equally often as was the internet. Another study of cancer patients in Hawaii who used CAM showed that participants were drawing on a similar range of information resources to those in the present study [8], but preferences for different modalities of health information varied according to ethnic group and educational level. The implications of these findings are twofold: first, the over-emphasis on regulation of internet information overlooks the fact that much information derived from other sources may be equally anecdotal or biased, and second, the assumption that the internet is the main source of information for patients may not necessarily be true.

One potential barrier to improving communication about CAM between patients and health professionals is likely to be the different standards of evidence used by patients and doctors in evaluating the effectiveness and safety of CAM therapies. Patients in the present study used a range of criteria to evaluate CAM therapies, usually favouring experiential evidence (patients' stories), rather than scientific evidence of effectiveness. This is similar to the findings of a Canadian study [30] and is in contrast to the regular calls for 'more scientific evidence' for CAM from members of the medical community.

Patients' decisions to use CAM may appear 'irrational' to clinicians, based as they are on limited scientific evidence, but patients are making meaningful and 'rational' decisions within their own frame of reference and value system. Debates about patients' apparently 'irrational' treatment choices do, however, pre-date the recent rise in popularity of CAM, and have tended to focus on the high rates of 'non-compliance' with or poor 'adherence' to conventional medical treatments [31]. A recent qualitative study of cancer patients' decisions to accept or refuse conventional treatment has highlighted the differing perspectives of doctor and patient [32]. In a similar way to the study reported here, doctors tended to apply a 'goal-oriented' rationality, contrasting with the 'value-oriented' rationality of patients. The authors concluded that clinicians' acceptance and understanding of an apparently 'irrational' decision is essential to establishing a better doctor-patient relationship in which the patient feels understood, respected and free to decide.

## Conclusion

This study reveals that male cancer patients who wish to use CAM would generally prefer to access information about CAM via the NHS. This concords with the recommendation of the NICE Guidelines for Supportive and Palliative Care that 'as a minimum, high quality information should be made available to patients about complementary therapies and services' [33]. For men in this study, finding out about CAM was part of a broader strategy of informing themselves about their condition and its treatment. Because of the distressing and life-threatening nature of their illness, cancer patients may be particularly susceptible to picking up information about their condition and how they might improve it. This could be used to positive benefit by the NHS in developing a strategy for the delivery of high quality CAM information and advice. The level and type of information would need to be graded in order to respect the needs of those who are likely to feel burdened by additional choices.

Men in this study were discerning in their evaluation of CAM information, offered rationales for their choices and often adopted a 'consumerist' approach to CAM treatment options. While health professionals often express concerns about patients being 'duped' by CAM providers offering a 'cure' at great expense, only a minority of men in this study chose to try a range of potentially 'curative' treatments at considerable financial cost. Information about such treatments was obtained through networks of acquaintances and sometimes through the internet. Website regulation and kite-marking is unlikely to deter these patients who wish to look beyond the boundaries of conventional medicine and choose alternative treatments precisely because they are outside the conventional healthcare system.

Our findings concord with those from a qualitative study in the USA which stressed the importance of finding a 'common ground for an open discussion in which physicians consider that scientific evidence is not all that counts in the life of an individual facing a serious disease' [4]. Without such open discussion, cancer patients using CAM may find themselves at odds with health professionals regarding their choices, or at least they may experience an indifferent response. This may have future consequences for care as it may discourage further disclosure of CAM use. The challenge for clinicians is to engage in open discussion with patients about CAM, to foster an environment of mutual trust that is likely to lead to better disclosure of CAM use rather than to perpetuate an atmosphere that may encourage covert, undisclosed use of CAM.

### Strengths and limitations of the study

Qualitative studies of information seeking such as this are less common than large-scale surveys and provide an opportunity for issues to be explored in more depth. This study also breaks new ground by focusing on men with a range of cancer types, who have been the focus of much less qualitative research in the field of CAM and cancer than women. This study aims to redress that balance. Men may formerly have been overlooked since surveys show that they use CAM less than women [34], they seek less information about CAM from a national cancer information service [12] and use the internet less than women for health information seeking [35]. The strategies that men adopt to access CAM information are therefore worthy of a specific study such as this one. A future study might directly address possible gender differences in accessing and evaluating CAM information.

As the study is qualitative, the findings are not necessarily representative of the views of all cancer patients but they may not be atypical and may well be transferable to cancer patients in similar settings in other parts of the UK. Care was taken to recruit patients across a wide range of ages, cancer types and disease stages, who came from a range of setting both within and outside the NHS and who are likely to present a wide spectrum of views and experiences. There were no participants from ethnic minority groups. The sample also comprised a majority of men with professional occupations, a finding typical of studies of CAM use. These findings add to the growing literature on health information seeking and make a unique contribution by focussing specifically on patients' search for and evaluation of CAM information.

## Competing interests

EAT is a consultant homeopathic physician at one of the recruitment settings used for this study. PT is the former Director of Education at one of the recruitment sites. The authors have no other competing interests.

## Authors' contributions

All authors contributed to the design, analysis and writing-up of this study. MAE was responsible for the day-to-day management and conduct of the study, conducted the interviews, led the analysis and produced the first draft of the manuscript. All authors have read and approved the final manuscript.

## Appendix 1: Glossary of 'complementary and alternative' treatments used in this study

**Nutrition: **Many followed a wheat-free, dairy-free, organic, vegan programme with nutritional supplements including vitamins, fish oils and selenium. Advice taken from a nutritionist, books or, occasionally, the internet. Following advice, some supplements were withheld during chemotherapy. A minority had experimented with a range of extreme 'anti-cancer' diets such as the Gerson Institute programme.

**'Mind – body therapies': **Healing, hypno-therapy, visualisation, relaxation, reiki, t'ai chi, qi gong and movement therapy.

**Homeopathy: **remedies prescribed by the consultant at the homeopathic clinic.

**Psychological therapies: **Individual and group counselling, positive affirmations, 'journeying' (exploring your soul journey using shamanic techniques), on-line peer support and the 'Health Creation Kit' (a self-help 'Cancer lifeline kit' developed by an Integrated Cancer Consultant).

**Physical and 'hands-on' therapies: **Acupuncture, massage, shiatsu, reflexology, aromatherapy, cranio-sacral therapy, kinesiology and exercise therapy.

### Herbal remedies

**Iscador: **fermented extract of mistletoe used to boost the immune system and provide anti-tumour properties.

**Bach Flower remedies: **diluted essences of flowers to re-balance the body's energy.

**Gingko biloba: **leaf extract used to improve blood circulation.

**Saw palmetto: **palm berry extract used for prostate symptoms.

**Essiac: **herbal tea containing burdock root, sheep sorrel, slippery elm and rhubarb used to treat cancer.

**Green tea: **used in China for 4,000 years to inhibit cancer cell growth.

**Apricot kernels**: a natural source of vitamin B17 used as an anti-cancer agent.

**Carctol: **an Indian ayurvedic compound used to treat cancer.

**Pycnogenol: **pine bark extract used as an anti-oxidant.

**Noni juice: **tropical plant extract used to support the immune system.

### Other 'alternative' therapies'

**Cyto-luminescent therapy: **whole body irradiation with light of a specific wavelength to selectively damage and eliminate tumour cells.

**Vitamin B17: **often given as an infusion and claimed to be anti-neoplastic.

**Psychic surgery: **an operation is performed with no scalpels or instruments. Body parts and masses and removed and no scar is left.

**Rife: **a treatment invented by Royal Rife in the 1930s to cure cancer using electronic frequencies.

**Parasite cleansing: **a variety of herbs used to cleanse the body of parasites.

**Colloidal silver: **Manufactured from silver, this treatment claims to be antiviral, antifungal and antibacterial.

## Appendix 2: Information provision and communication about CAM with health professionals

### Lack of interest in CAM shown by clinicians

'Q: Do the oncologists know that you're taking essiac?

They're not interested. They're not remotely interested in what I'm taking or not taking ... I've told them and they say mmm mmm yes they're not interested in anything' *[age 42, metastatic thymic cancer]*

### Patients' desire for NHS to offer CAM information

'I've mentioned it to the doctors but they don't seem very forthcoming, not on the National Health anyway, they don't seem to want to .... Really I would go and have a go if someone recommended something, ... there should be leaflets in doctor's surgeries or hospitals so you can look at it, like in the chemotherapy if there was leaflets on the walls saying why don't you try this and that just to help you get through ... I'm sure there must be things that could probably help with the side effects of chemotherapy because there's a lot of them to be honest, lots of things you can get' *[age 43, locally advanced colo-rectal cancer]*

'Down at the oncology centre they don't really mention it [the homeopathic hospital] but they can give you stuff what can help your body and help your immune system that's what you need the nurse to say' *[age 43, metastatic colo-rectal cancer]*

## Appendix 3: The role of family and friends

### Female relatives/partners

'My ex-girlfriend is a shiatsu practitioner .... So I spoke to her about it, she plugged me with books which is her thing, her thing's books, information, information, information she loves all that and she reads and reads ferociously and ah I'm the opposite you know I want to kind of I want to get information personally' *[age 47, early stage leukaemia]*

### Friends

'We were looking for a doctor and we just got talking to one of our friends who mentioned him (GP homeopath), we went to see him and he was such a lovely man and he had personality, you had confidence in him really, I wasn't worried if he used alternative medicines, 'cos some doctors just give you a packet of pills and you're gone' *[age 54, prostate cancer in remission]*

## Appendix 4: The role of the internet

### Discerning use of the internet

'Once I found one [website] that I was getting quite a lot of information from I didn't go much farther than that ... I didn't have, I'm not one of those that goes to every site' *[age 58, metastatic prostate cancer]*

'I've always been a little put off by the sort of web-sites that don't seem to be very based in logic or evidence ... I want to be presented with some evidence that something works other than just personal comments' *[age 42, metastatic thymic cancer]*

### Internet to follow up suggestions

'People have mentioned oh have you tried this, or have you tried this ... so then I use the internet to look up these things that people mention .. so I actually found the internet in that sense brilliant' *[age 43, recurrent bone cancer]*

### Non-use of the internet

'I don't [use the internet] because the information's supposed to be given to you at the sharp end isn't it, the hospital .... Well I've got a computer, but I don't particularly chase the internet .... I'm not particularly computer oriented I'm surrounded by computers but I'm not the kind to want to know things about it' *[age 57, metastatic colo-rectal cancer]*

## Appendix 5: 'Traditional' sources of information used by participants

### Books and articles

'General books, things in the newspapers, I did read a few things when we first went to [homeopathic GP]. I did read a few articles. I went to the library to read a few things about alternative medicines .... You have to be sceptical at first .... But I have done you know I've read a few books now. It did make me think a bit differently' *[age 54, prostate cancer in remission]*

### Leaflets

'I picked up a sheet from their church [Spiritualist] and there was an advertisement in there for a Finnish bark product .... And when I was in the newspaper I used to get a lot of books to review and one of them was a book by another journalist who had gone to South America to investigate these wonder cures .... And when I went to the [private cancer charity] I asked I wanted a book about the mental attitude' *[age 79, metastatic prostate cancer]*

### Magazines

'There are these buildings which offer complementary support and therapies [to cancer patients] and so they're attached to an oncology department.

Q: So how did you find out about them?

Um, when I was at the [GP practice], there was a, I was reading a copy of Vogue or something called Marie, I think it was Vogue, ah from last year and they had a big article about it' *[age 41, metastatic lung cancer]*

## Appendix 6: Patients as pro-active seekers or passive recipients of information

### Pro-active by choice

'I suppose it's down to the individual to sort of do their own research and make up their own minds about what they want to do' *[age 60, locally advanced lung cancer]*

### Passive by choice

'He [oncologist] just, it was very caring actually you know he knew that we weren't ones to, we're quite passive people actually in a sense, me and my wife, we're not ones which want to, you know, so many questions we want all the answers, we just knew we want the information there, we could just go away and digest it' *[age 49, metastatic colo-rectal cancer]*

## Appendix 7: Patients' criteria for evaluating CAM

### Historical use of CAM

'No because if they have been efficacious for centuries whilst there might be an interest in trying to measure them scientifically, like Chinese techniques ... there is no doubt it works which is an amazing thing ... the history of acupuncture it goes back over three thousand years' *[age 73, prostate cancer in remission]*

### Plausibility

'Yes, and ...um... [pause] they do kind of holding hands business and yes ... I don't go for that at all [laughing] you know, and ...um... I don't think you can cure cancer just by saying 'I haven't got it', you know it ..um ...I don't think that would work .. no ...um... anything that smacks of kind of mysticism or spiritualism or that it's to me no it's a waste of time I just cannot accept that' *[age 83, locally advanced prostate cancer]*

### Belief and trust in therapies and their providers

'I was once invited to go to the cranio-sacral therapist .... My wife knows him .... I found it scary, the techniques, putting pressure points on the skull and you, the patient, don't know what's happening. There was a 'guru atmosphere' to this guy. I couldn't take it'

'Things like acupuncture I wouldn't try .... You should see the charts! It depends how desperate you were, for example if the clinical system had given up on you. I wouldn't touch reflexology with a barge-pole if it wasn't for my daughter doing it. But I wouldn't go along with the medics who poo-poo alternatives .... But the way out stuff I would need proof, or a person to recommend it who I could trust, like the person who recommended Dr L. A certificate on the wall is not enough' *[age 66, prostate cancer in remission]*

### Scientific evidence

'If you wait until something is absolutely tried and tested and guaranteed to work but in fact nothing is guaranteed to work ... Um you know it all depends on the individual and I think you know one thing that does convince me is you know if one person is able to recover or have a full remission from cancer without medical treatment, that proves the body can have the capacity to do it' *[P34 age 53, locally advanced non-Hodgkin's lymphoma]*

## Pre-publication history

The pre-publication history for this paper can be accessed here:


